# ﻿Four new species of *Laelius* Ashmead (Hymenoptera, Bethylidae) from Korea with an updated key to species in the Eastern Palaearctic region

**DOI:** 10.3897/zookeys.1213.121630

**Published:** 2024-09-27

**Authors:** Jongok Lim, Seunghwan Lee

**Affiliations:** 1 Department of Life and Environmental Sciences, College of Agriculture and Food Sciences, Wonkwang University, Iksan, Jeonbuk Province, Republic of Korea Wonkwang University Iksan Republic of Korea; 2 Institute of Life Science and Natural Resources, Wonkwang University, Iksan, Jeonbuk Province, Republic of Korea Seoul National University Seoul Republic of Korea; 3 Laboratory of Insect Biosystematics, Department of Agricultural Biotechnology, Seoul National University, Seoul, Republic of Korea Wonkwang University Iksan Republic of Korea; 4 Research Institute of Agriculture and Life Sciences, Seoul National University, Seoul, Republic of Korea Seoul National University Seoul Republic of Korea

**Keywords:** Asia, Epyrinae, flat wasps, identification key, new species, taxonomy

## Abstract

The genus *Laelius* Ashmead, 1893 (Hymenoptera, Bethylidae) is a cosmopolitan bethylid genus with 68 valid species distributed across most zoogeographic regions worldwide. This taxonomic study on Korean species of *Laelius* has led to the description of four new species, namely *L.afores***sp. nov.**, *L.atratus***sp. nov.**, *L.sulcatus***sp. nov.**, and *L.tricuspis***sp. nov.** Additionally, illustrations of the diagnostic characteristics of each species are provided, along with an updated key for 11 *Laelius* species from the Eastern Palaearctic region.

## ﻿Introduction

The genus *Laelius* Ashmead, 1893 (Hymenoptera, Bethylidae) is a cosmopolitan genus belonging to the subfamily Epyrinae. Currently, it comprises 68 valid species distributed across all zoogeographic regions, except for the Oceanian region as suggested by Holt et al. (2013). Three extinct fossil species have been recorded from Baltic and Rovno amber (Azevedo et al. 2018; Colombo et al. 2021). Approximately 33% (22 species) of the species were described from the Palaearctic region, with seven species reported from Eastern Asia.

Taxonomic information on *Laelius* species in the European Palaearctic region, mainly from Finland and Sweden, was provided by Vikberg and Koponen (2005). *Laeliussinicus* Xu, He & Terayama in the Eastern Palaearctic region was described by Xu et al. (2003), and Terayama (2006) published a book on Japanese Bethylidae, which included four *Laelius* species from Japan. *Laeliusyamatonis* Terayama, 2006 was later recorded from South Korea by Lim et al. (2010a), and Lim et al. (2010b) provided a key to the Eastern Palaearctic species with a description of *L.jilinensis* Lim & Lee, 2010 from China. Currently, only one species, *L.yamatonis* Terayama, 2006, has been recorded in South Korea (Lim et al. 2010a).

*Laelius* species can be distinguished by the presence of thick black setae on the body and wings, a projected median clypeal lobe, and complete occipital carinae (Azevedo et al. 2018). The presence and relative length of the carinae on the metapostnotum and the metapectal-propodeal disc, as well as the relative length of the 2r-rs&Rs vein of the forewings, are commonly used in taxonomic and systematic studies on *Laelius* (Marques Jr. et al. 2023).

*Laelius* species parasitize larvae of various families, including Dermestidae and Scolytinae (Coleoptera), and Glossinidae (Diptera) (Vance 1932; Mertins 1980; Azevedo et al. 2018). Furthermore, *Laelius* species have been utilized for biological control purposes, as documented in many reports (Barbosa and Azevedo 2011).

This manuscript describes four new species of *Laelius* from South Korea, providing illustrations of their diagnostic characteristics and an updated taxonomic key for 11 *Laelius* species from the Eastern Palaearctic region.

## ﻿Materials and methods

All materials examined were collected using Malaise traps from the northern and southern regions of South Korea. The abbreviations for collection localities in Korea are as follows: **GW**, Gangwon-do; **JN**, Jeollanam-do; **JJ**, Jeju-do. Detailed information on provincial localities in South Korea can be found in Lim et al. (2011).

The abbreviations for biometric measurements used in the text are as follows: **AOL**, the minimum distance between the anterior ocellus and the posterior ocellus; **DAO**, the diameter of the anterior ocellus; **HE**, the height (maximum length) of the eye in lateral view; **LFW**, the maximum length of the forewing; **LH**, the length of the head, from the apical margin of the clypeus to the posterior margin of the head in full dorsal view; **POL**, the distance between the posterior ocelli in full dorsal view; **VOL**, the vertex-ocular line, which is the distance between the top of the eye and the vertex line; **WE**, the minimum width of the eye in lateral view; **WF**, the width of the frons, the minimum distance between the eyes in full dorsal view; **WH**, the maximum width of the head in full dorsal view; **WOT**, the width of the ocellar triangle, including the width of the posterior ocelli.

Terms describing integument sculptures followed Eady (1968) and Harris (1979). Additionally, general morphological terms followed Azevedo et al. (2018), Lanes et al. (2020), and terms regarding mesoplueral structures followed Brito et al. (2021).

The specimens were examined under a Leica M205 C stereomicroscope (Leica Microsystems, Solms, Germany), and images were captured with a Dhyana 400D camera (TUCSEN CMOS, Fujian, China) attached to a Leica M205C. Multi-stacked images were produced using the Delta Multifocus ver. 24 program (Delta, South Korea) and Helicon Focus ver. 8.2.2 software (HeliconSoft, Ukraine). The final images were edited using Adobe Photoshop 2021 (Adobe Systems, Inc., San Jose, CA, USA).

The examined specimens were deposited at the Laboratory of Insect Taxonomy and Ecology at Wonkwang University (W-LITE), Iksan, Republic of Korea.

## ﻿Systematic accounts

**Family Bethylidae Haliday, 1839**

**Subfamily Epyrinae Kieffer, 1914**

### ﻿Genus Laelius

Taxon classificationAnimaliaHymenopteraBethylidae

Ashmead, 1893

1262AE06-AFDC-5F13-9220-B4D320E36D36

Laelius Ashmead, 1893. Bull. U.S. Nat. Mus., 45: 50. Type-species: Laeliustrogodermatis Ashmead, 1893.
Paralaelius Kieffer, 1905. Ann. Soc. Sci. Bruxelles, 29: 129. Type-species: Laeliuspedatus (Say, 1836).
Allepyris Kiffer, 1905. Ann. Soc. Sci. Burxelles, 29: 106. Type-species: Allepyrismicroneura (Kieffer, 1905).
Prolaelius Kieffer, 1905. Type-species: Paralaeliusfirmipennis (Cameron, 1905).

#### Diagnosis.

The genus *Laelius* can be distinguished from other genera by having the mesoscuto-scutellar suture with an evident sulcus and thick black setae on the body (Colombo et al. 2022).

### ﻿ Laelius afores

Taxon classificationAnimaliaHymenopteraBethylidae

Lim sp. nov.

E6A4995F-3942-5858-ACD8-F711278B85D3

https://zoobank.org/6742C3C5-B66C-4E1C-8EDF-1F9A874FF85A

Fig. 1A–E

#### Description.

Holotype (female). Body length 2.61 mm; LFW 1.48 mm.

***Color*.** Head black; mandible dark castaneous except apical half-light castaneous; antenna dark castaneous except scape and pedicel light castaneous in dorsal view. Mesosoma black; legs light castaneous except basal half of procoxae and metafemora castaneous; wings hyaline, tegula light castaneous, veins pale castaneous. Metasoma black.

#### Morphology.

**Figure 1.**
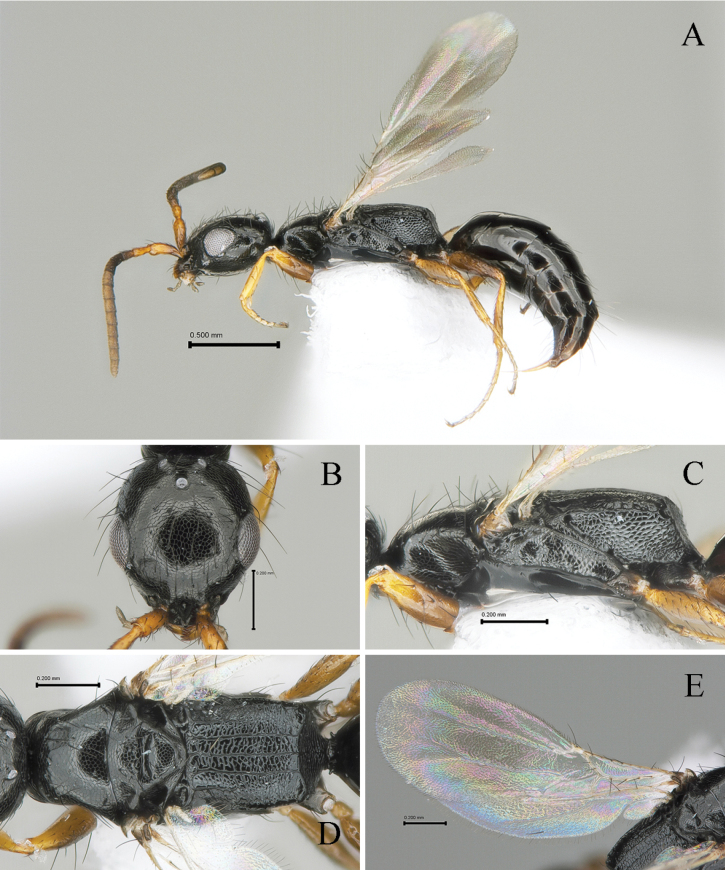
*Laeliusafores* Lim, sp. nov., holotype (female) **A** habitus in lateral view **B** head in dorsal view **C** mesosoma in lateral view **D** ditto, in dorsal view **E** forewing in dorsal view. Scale bars: 0.50 mm (**A**); 0.20 mm (**B–E**).

***Head*** (Fig. 1A, B). 1.1× as long as wide with broadly outcurved vertex crest in dorsal view in dorsal view (Fig. 1B). Ventral and lateral surface with suberect long setae, some setae longer than LE. Mandible with four apical teeth; two uppermost teeth small, 3^rd^ tooth from top most thick and one ventralmost sharpened. Clypeus short, broadly rounded with one median small blunt tooth, median area longitudinally elevated (Fig. 1B). First five antennomeres in ratio of 2.5: 1.7: 2.5: 1.0: 1.0 in length; scape, pedicel and flagellomere III–V and XI 2.7, 2.4, 3.3, 1.2, 1.3 and 1.3× as long as wide, respectively. Frons coriaceous with sparse punctures; frontal line absent. Vertex slightly outcurved and round marginally. WF 1.9× LE, WF 0.7× WH. Compound eye 0.19 mm long without setae. Medioccipito-genal suture present. Occipital carina complete. LE 1.0× OOL, WF 2.3× WOT. Anterior angle of ocellar triangle obtuse, POL 1.3× AOL, OOL 1.3× WOT (Fig. 1B).

***Mesosoma*** (Fig. 1A, C–E). Dorsal pronotal area coriaceous as head, 0.5× as long as wide, trapezoidal, humeral angle obtuse; punctures very shallow and sparse (Fig. 1D); posterior pronotal sulcus absent. Mesoscutum coriaceous as head, pronotum with short and thin setae; notaulus absent; parapsidal signum thin, reaching posterior margin of mesoscutum (Fig. 1D). Mesoscutellar disc coriaceous; mesoscuto-scutellar ridge slightly posterad at each side (Fig. 1D). Metapectal-propodeal disc 1.1× as long as wide; metapostnotal median carina and first metapostnotal lateral carinae complete, strong, completely extending transverse posterior carina of metapectal-propodeal complex; second metapostnotal lateral carina extending one-fourth of disc; paraspiracular carina distinct, presented on distal half of disc, reaching transverse posterior carina of metapectal-propodeal complex; transverse posterior carina of metapectal-propodeal complex complete and concave in dorsal view. Propodeal declivity reticulate without median carina (Fig. 1D). Mesopleuron with mesepimeral sulcus; subalar fovea oval and closed; lower mesopleural fovea opened (Fig. 1C). Femora and tibia with long setae on outer surface, getting short to tarsomeres. Tegula without some erect setae. Subcostal vein (Sc_2_v), median vein (M_2_v) and anal vein (A_2_v) with long setae; second radial cross vein and radial sector vein of fore wing (2r-rs&Rs_2_v) 0.9 mm long, 0.8× as long as Rs+M_2_v. First median cell of fore wing and second median cell of fore wing with few short hairs (Fig. 1E). Hind wing with three distal hamuli.

***Metasoma*** (Fig. 1A). Tergum I and II largely smooth, polished without distinct long erect hairs; remaining terga with microreticulation on anterior half; terga III–VI with transverse sparse setae line on dorsal surface.

#### Material examined.

***Holotype*.** Female. Seoul National University, Sinlim, Gwanak, Seoul, South Korea. 6.iv.2020. Deok-Young Park leg. (W-LITE).

#### Distribution.

South Korea (Seoul).

#### Etymology.

The specific epithet afores refers to the absence of notaulus on the mesoscutum and median carina on the propodeum declivity.

#### Remarks.

The species is similar to *L.jilinensis* Lim & Lee, 2010 from China by ‘overall color of body and appendages; head as long as wide with broadly outcurved vertex in dorsal view; mandible with four teeth; clypeus short with one median small blunt tooth on anterior margin; compound eye without setae; metapectal-propodeal disc as long as wide with metapostnotal median carina and first- and second metapostnotal lateral carina’. However, *L.afores* Lim, sp. nov. is distinguished from *L.jilinensis* by ‘WF 1.9× LE (WF 1.4× LE in *L.jilinensis*); LE 1.0× OOL (LE 1.2× LE *in L.jilinensis*); dorsal pronotal area 0.5× as long wide (0.8× as long as in *L.jilinensis*); mesoscutum without notaulus (notaulus absent in *L.jilinensis*); propodeal declivity without median longitudinal carina (median longitudinal carina present in *L.jilinensis*)’.

### ﻿ Laelius atratus

Taxon classificationAnimaliaHymenopteraBethylidae

Lim sp. nov.

BD170392-4146-5834-AAB3-4E666EF8FE69

https://zoobank.org/C90ABCF0-CAD1-4B48-98F4-0029C44130E3

Fig. 2A–E

#### Description.

Holotype (female). Body length 3.05 mm; LFW 1.67 mm.

***Color*.** Head black; mandible light castaneous; antenna dark castaneous except apical half of scape, pedicel and basal flagellomere I light castaneous in dorsal view. Mesosoma black; legs light castaneous except coxa and femora dark black; wings hyaline, tegula light castaneous, veins pale castaneous. Metasoma black.

#### Morphology.

**Figure 2.**
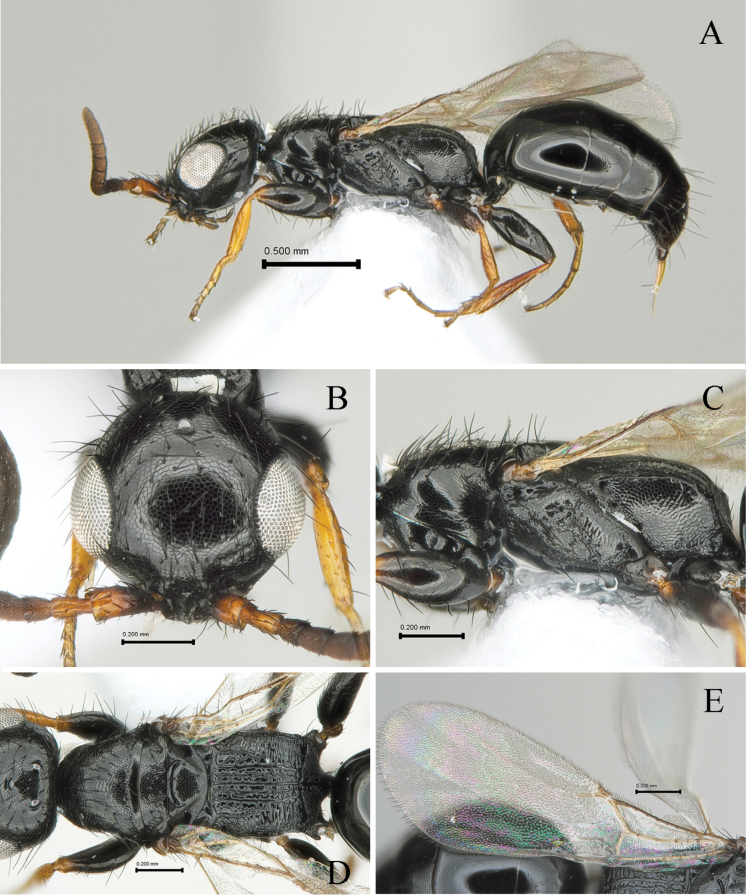
*Laeliusatratus* sp. nov., holotype (female) **A** habitus in lateral view **B** head in dorsal view **C** mesosoma in lateral view **D** ditto, in dorsal view **E** forewing in dorsal view. Scale bars: 0.50 mm (**A**); 0.20 mm (**B–E**).

***Head*** (Fig. 2A, B). 1.1× as long as wide with slightly outcurved vertex crest in dorsal view (Fig. 2B). Ventral and lateral surface with erect or suberect setae. Mandible with four apical teeth; two uppermost teeth small, 3^rd^ tooth from top most thick and one ventralmost sharpened. Clypeus short, anterior margin straight with one median very minute triangle tooth; median area longitudinally elevated (Fig. 2B). First five antennomeres in ratio of 3.1: 1.3: 1.0: 1.1: 1.1 in length; scape, pedicel and flagellomere III–V and XI 2.5, 1.2, 1.4, 1.2, 1.0 and 1.5× as long as wide, respectively. Frons coriaceous with sparse punctures, polished; frontal line absent. Vertex slightly outcurved and round marginally. WF 1.4× LE. WF 0.7× WH. Compound eye 0.29 mm long without setae in lateral view. Medioccipito-genal suture present. Occipital carina complete. LE 1.4× OOL, WF 2.2× WOT. Anterior angle of ocellar triangle slightly obtuse, POL 1.2× AOL, OOL 1.1× WOT (Fig. 2B).

***Mesosoma*** (Fig. 2A, C–E). Dorsal pronotal area coriaceous as head, 0.6× as long as wide, trapezoidal, humeral angle obtuse; punctures shallow and sparse as head (Fig. 2D); posterior pronotal sulcus absent. Mesoscutum coriaceous as head, pronotum with short and thin setae; notaulus distinct, short on distal half of mesoscutum; parapsidal signum deep and reaching posterior margin of mesoscutum (Fig. 2D). Mesoscutellar disc coriaceous; mesoscuto-scutellar ridge posterad at each side (Fig. 2D). Metapectal-propodeal disc 0.9× as long as wide; metapostnotal median carina and second metapostnotal lateral carinae strong, completely extending transverse posterior carina of metapectal-propodeal complex; first metapostnotal lateral carinae weak, completely extending transverse posterior carina of metapectal-propodeal complex; third metapostnotal lateral carinae extending one-third of disc; paraspiracular carinae distinct, completely reaching transverse posterior carina of metapectal-propodeal complex; transverse posterior carina of metapectal-propodeal complex complete and concave in dorsal view (Fig. 2D). Propodeal declivity reticulate with median carina (Fig. 2D). Mesopleuron with mesepimeral sulcus; subalar fovea elongated and closed; lower mesopleural fovea opened (Fig. 2C). Femora and tibia with long setae on outer surface, getting short to tarsomeres. Tegula with some erect setae. Subcostal vein (Sc_2_v), median vein (M_2_v) and anal vein (A_2_v) with long setae; second radial cross vein and radial sector vein of fore wing (2r-rs&Rs_2_v) 0.1 mm long, 0.8× as long as Rs+M_2_v. First median cell of fore wing and second median cell of fore wing with few short hairs (Fig. 2E). Hind wing with three distal hamuli.

***Metasoma*** (Fig. 2A). Tergum I and II largely smooth, polished without distinct long erect hairs; remaining terga with microreticulation on anterior half; terga III–VI with transverse sparse setae line on dorsal surface.

#### Material examined.

***Holotype***: Female. Aguala Hotel Dorm., 1388. Hyeopjae. Hallim, JJ, South Korea. 33°23'58.8"N, 126°14'57.0"E, Malaise trap, 17.vii–20.viii.2017, Sanghyeok Nam leg. (W-LITE); ***paratype***: Female. same collection data as holotype (W-LITE).

#### Distribution.

South Korea (JJ).

#### Etymology.

The specific epithet atratus refers to the submedian carinae parallel to the median discal carinae.

#### Remarks.

The species is similar to *L.nigrofemoratus* Terayama, 2006 from Japan by ‘color of head, mesosoma and metasoma; head as long as wide with convex posterior margin in dorsal view; metapectal-propodeal complex with one metapostnotal median carina and three pairs of metapostnotal lateral carinae’. However, *L.atratus* Lim, sp. nov. is distinguished from *L.nigrofemoratus* Terayama by ‘mandible with four teeth (mandible with five teeth in *L.nigrofemoratus*); legs reddish brown except coxa and femora black (legs reddish brown including coxa in *L.nigrofemoratus*); first metapostnotal lateral carinae parallel to metapostnotal median carina (first metapostnotal lateral carinae extending to metapostnotal median carina near transverse posterior carina of metapectal-propodeal complex in *L.nigrofemoratus*)’.

### ﻿ Laelius sulcatus

Taxon classificationAnimaliaHymenopteraBethylidae

Lim sp. nov.

60D4CB9D-C13A-50C4-A755-C4FB714B7DC8

https://zoobank.org/DC81C584-C302-468B-BA97-5315024018C8

Fig. 3A–E

#### Description.

Holotype (female). Body length 4.35 mm; LFW 2.46 mm.

***Color*.** Head black; mandible castaneous; antenna dark castaneous except scape, pedicel, flagellomere I castaneous in dorsal view. Mesosoma black; legs castaneous except coxa dark castaneous; wings hyaline, tegula light castaneous, veins pale castaneous. Metasoma black.

#### Morphology.

**Figure 3.**
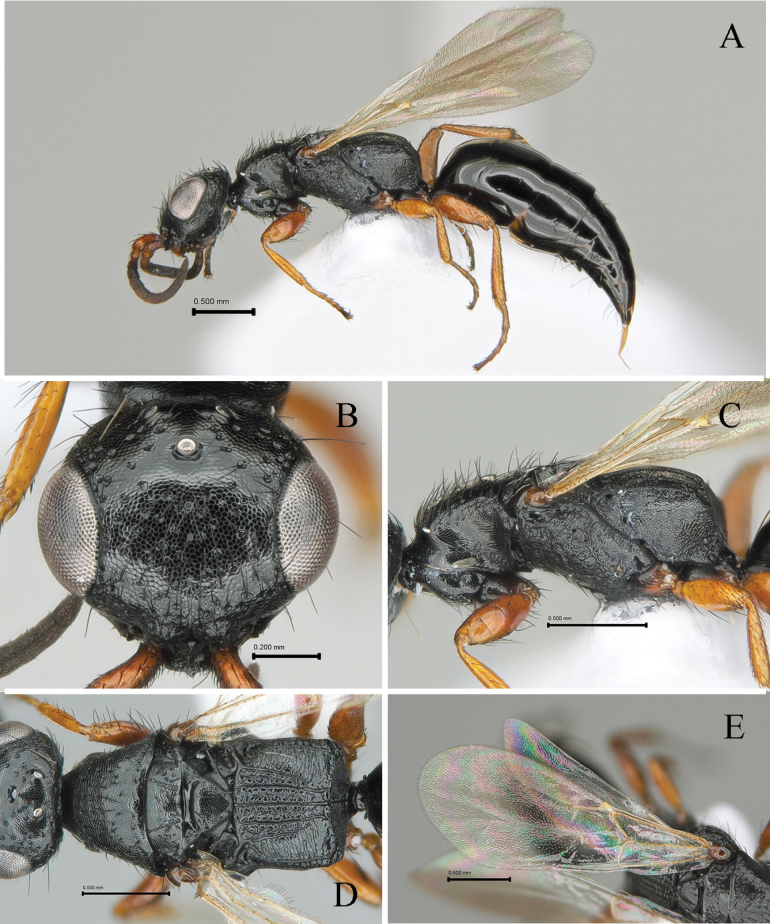
*Laeliussulcatus* sp. nov., holotype (female) **A** habitus in lateral view **B** head in dorsal view **C** mesosoma in lateral view **D** ditto, in dorsal view **E** forewing in dorsal view. Scale bars: 0.50 mm (**A**); 0.20 mm (**B–E**).

***Head*** (Fig. 3A, B). 1.1× as long as wide with broadly outcurved vertex crest in dorsal view in dorsal view (Fig. 3B). Ventral and lateral surface with suberect long setae, each seta shorter than LE. Mandible with five apical teeth; three uppermost teeth small, 4^th^ tooth from top most thick and one ventralmost long and sharpened. Clypeus short, broadly rounded with one median small blunt tooth, median area longitudinally elevated (Fig. 3B). First five antennomeres in ratio of 3.0: 1.4: 1.2: 1.0: 1.0 in length; scape, pedicel and flagellomere III–V and XI 2.5, 1.7, 1.3, 1.0, 1.0 and 1.8× as long as wide, respectively. Frons coriaceous with sparse big punctures; frontal line absent. Vertex slightly outcurved and round marginally. WF 1.3× LE, WF 0.7× WH. Compound eye 0.40 mm long without setae. Medioccipito-genal suture present. Occipital carina complete. LE 1.7× OOL, WF 2.1× WOT. Anterior angle of ocellar triangle obtuse, POL 1.4× AOL, OOL 0.9× WOT (Fig. 3B).

***Mesosoma*** (Fig. 3A, 3C–E). Dorsal pronotal area coriaceous as head, 0.5× as long as wide, trapezoidal, humeral angle obtuse; punctures shallower and smaller than punctures on head (Fig. 3D); posterior pronotal sulcus absent. Mesoscutum coriaceous as head, pronotum with short and thin setae; notaulus long, distinct; parapsidal signum thick, present distal half, reaching posterior margin of mesoscutum (Fig. 3D). Mesoscutellar disc coriaceous; mesoscuto-scutellar ridge wide and deep, posterad at each side (Fig. 3D). Metapectal-propodeal disc 0.7× as long as wide; metapostnotal median carina and second metapostnotal lateral carinae distinct, strong, extending transverse posterior carina of metapectal-propodeal complex; second metapostnotal lateral carinae extending distal one fifth of disc; paraspiracular carinae distinct, reaching transverse posterior carina of metapectal-propodeal complex; transverse posterior carina of metapectal-propodeal complex complete and concave in dorsal view (Fig. 3D). Propodeal declivity reticulate with distinct median carina (Fig. 3D). Mesopleuron with mesepimeral sulcus; subalar fovea oval and closed; lower mesopleural fovea opened (Fig. 3C). Femora and tibia with long setae on outer surface, getting short to tarsomeres. Tegula with some erect setae. Subcostal vein (Sc_2_v), median vein (M_2_v) and anal vein (A_2_v) with long setae; second radial cross vein and radial sector vein of fore wing (2r-rs&Rs_2_v) 0.3 mm long, 1.0× as long as Rs+M_2_v. First median cell of fore wing and second median cell of fore wing with few short hairs (Fig. 3E). Hind wing with three distal hamuli.

***Metasoma*** (Fig. 3A). Tergum I and II largely smooth, polished without distinct long erect hairs; remaining terga with microreticulation on anterior half; terga III–VI with transverse sparse setae on dorsal surface.

#### Material examined.

***Holotype***: Female. 854 Hangye-ri, Buk-myeon, Inje-gun, GW, South Korea. 38°08'46.5"N, 128°15'47.5"E, Malaise trap, 11–29.vi.2017, Sanghyeok Nam leg. (W-LITE); ***paratypes***: 2 Females, Forahn House, 703 Ongpo-ri, Hanlim-eub, JJ, South Korea. 33°12'51.1"N, 126°15'04.0"E, Malaise trap, 16.v.2018, Sanghyeok Nam leg. (W-LITE); Female, Aguala Hotel Dorm., 1388. Hyeopjae. Hallim, Jeju, South Korea. 33°23'58.8"N, 126°14'57.0"E, Malaise trap, 17.vii–20.viii.2017, Sanghyeok Nam leg. (W-LITE).

#### Distribution.

South Korea (GW, JJ).

#### Etymology.

The specific epithet sulcatus refers to the distinctly developed notaulus on the mesoscutum.

#### Remarks.

The species is similar to *L.yamatonis* Terayama, 2006 from Korea and Japan by ‘head slight longer than wide with convex posterior margin in dorsal view; mandible with five teeth; clypeus broadly rounded; pedicel about 1.7–1.8 times as long as wide; dorsal pronotal area 0.5× as long as wide; metapectal-propodeal complex with one metapostnotal median carina and two pairs of metapostnotal lateral carinae ‘. However, *L.sulcatus* Lim, sp. nov. is distinguished from *L.yamatonis* Terayama by ‘ LE 1.7× OOL (LE 1.3× OOL in *L.yamatonis*; mesoscutum with distinct notalulus (mesoscutum without notaulus in *L.yamatonis*); second metapostnotal lateral carinae reaching basal three fourth of metapostnotal-propodeal disc (second metapostnotal lateral carinae reaching basal one-fourth of metapostnotal-propodeal disc in *L.yamatonis*)’.

### ﻿ Laelius tricuspis

Taxon classificationAnimaliaHymenopteraBethylidae

Lim sp. nov.

F28981DC-ED76-5453-AA17-3CE0CC9AFAC8

https://zoobank.org/7D6FE7CC-CB2A-47A3-8D3E-E6723B42DA01

Fig. 4A–E

#### Description.

Holotype (female). Body length 2.57 mm; LFW 1.61 mm.

***Color*.** Head black; basal half of mandible dark castaneous and apical half-light castaneous; antenna castaneous except basal two thirds dark castaneous in dorsal view. Mesosoma black; legs castaneous except coxa and femora dark castaneous; wings hyaline, tegula light castaneous, veins pale castaneous. Metasoma black.

#### Morphology.

**Figure 4.**
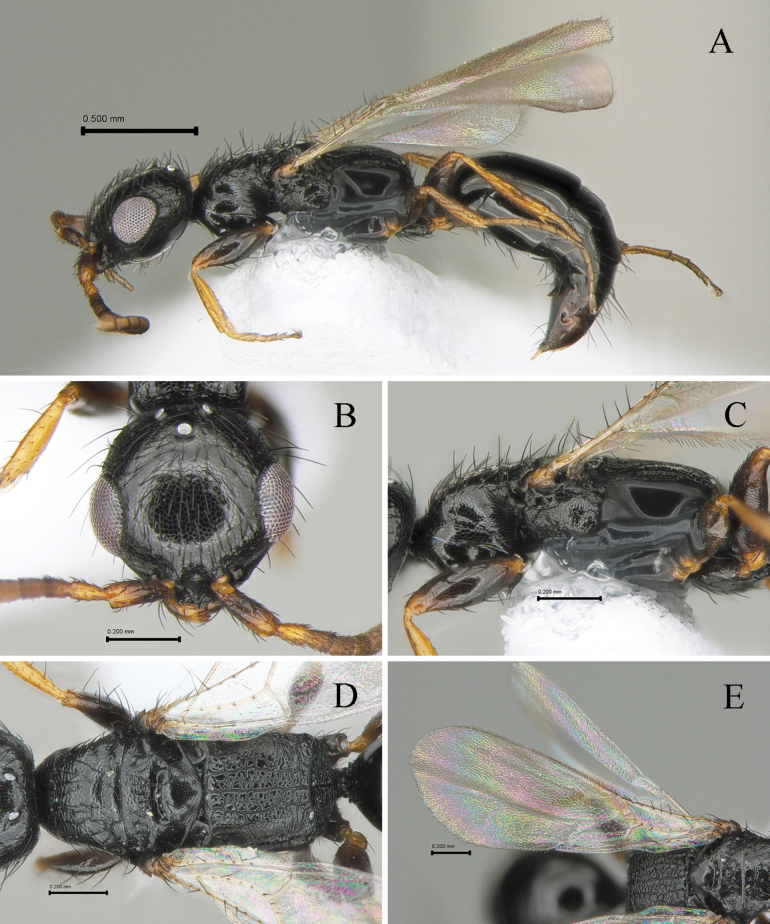
*Laeliustricuspis* sp. nov., holotype (female) **A** habitus in lateral view **B** head in dorsal view **C** mesosoma in lateral view **D** ditto, in dorsal view **E** forewing in dorsal view. Scale bars: 0.50 mm (**A**); 0.20 mm (**B–E**).

***Head*** (Fig. 4A, B). 1.0× as long as wide with slightly convex posterior margin in dorsal view (Fig. 4B). Ventral and lateral surface with suberect long setae, some setae as long as LE. Mandible with four apical teeth; two uppermost teeth small, 3^rd^ tooth from top most thick and one ventralmost sharpened. Clypeus short, anterior margin straight with one median small blunt tooth, median area weakly elevated (Fig. 4B). First five antennomeres in ratio of 3.5: 1.6: 1.1: 1.1: 1.0 in length; scape, pedicel and flagellomere III–V and XI 3.3, 2.0, 1.2, 1.0, 1.0 and 1.5× as long as wide, respectively. Frons coriaceous with sparse punctures; frontal line absent. Vertex slightly outcurved and round marginally. WF 1.6× LE, WF 0.7× WH. Compound eye 0.24 mm long without setae. Medioccipito-genal suture present. Occipital carina complete. LE 1.3× OOL, WF 2.4× WOT. Anterior angle of ocellar triangle obtuse, POL 1.4× AOL, OOL 1.1× WOT (Fig. 4B).

***Mesosoma*** (Fig. 4A, C–E). Dorsal pronotal area coriaceous as head, 0.5× as long as wide, trapezoidal, humeral angle obtuse; punctures very shallow and sparse (Fig. 4D); posterior pronotal sulcus present. Mesoscutum coriaceous as head, pronotum with short and thin setae; notaulus weak, present on distal half of mesoscutum; parapsidal signum short, present on distal one third of mesoscutum (Fig. 4D). Mesoscutellar disc coriaceous; mesoscuto-scutellar ridge slightly posterad at each side (Fig. 4D). Metapectal-propodeal complex 1.0× as long as wide; metapostnotal median carina and first metapostnotal lateral carinae distinct, completely extending transverse posterior carina of metapectal-propodeal complex; median area rugulose; submedian area strigate; paraspiracular carinae distinct, reaching transverse posterior carina of metapectal-propodeal complex; transverse posterior carina of metapectal-propodeal complex complete and concave in dorsal view (Fig. 4D). Propodeal declivity reticulate with median carina (Fig. 4D). Mesopleuron with mesepimeral sulcus; subalar fovea oval and closed; lower mesopleural fovea opened (Fig. 4C). Femora and tibia with long setae on outer surface, getting short to tarsomeres. Tegula without some erect setae. Subcostal vein (Sc_2_v), median vein (M_2_v) and anal vein (A_2_v) with long setae; second radial cross vein and radial sector vein of fore wing (2r-rs&Rs_2_v) 0.2 mm long, 1.0× as long as Rs+M_2_v. First median cell of fore wing and second median cell of fore wing with few short hairs (Fig. 4E). Hind wing with three distal hamuli.

***Metasoma*** (Fig. 4A). Tergum I and II largely smooth, polished without distinct long erect hairs; remaining terga with microreticulation on anterior half; terga III–VI with transverse sparse setae line on dorsal surface.

#### Material examined.

***Holotype***. Female. Is. Geumho, Sani-myeon, Haenam-gun, JN, South Korea. 34°41'19"N, 126°21'19"E, Malaise trap, 1–27.vi.2023, Jongok Lim leg. (W-LITE).

#### Distribution.

South Korea (JN).

#### Etymology.

The specific epithet tricuspis refers to the long and distinct three metapectal-propodeal carinae (one metapostnotal median carina and one pair of metapostnotal lateral carinae).

#### Remarks.

The species is similar to *L.jilinensis* Lim & Lee, 2010 from China by ‘overall color of body and appendages; head as long as wide with convex posterior margin in dorsal view; mandible with four teeth; clypeus short with one median small tooth medially on straight anterior margin; compound eye without setae’. However, *L.tricuspis* Lim, sp. nov. is distinguished from *L.jilinensis* by ‘scape 3.5× as long as flagellomere III (scape 2.8× as long as flagellomere III in *L.jilinensis*); WF 2.4× WOT (WF 2.2× WOT in *L.jilinensis*); pronotal disc 0.5× as long as wide (0.8× as long as wide in *L.jilinensis*); metapectal-propodeal complex with three metapostnotal lateral carinae (five metapostnotal lateral carinae present in *L.jilinensis*)’.

### ﻿Key to the *Laelius* species from the Eastern Palaearctic region

**Table:** 

1	POL wider than OOL	***L.antropovi* Gorbatovsky, 1995 (Far Eastern Russia)**
–	POL narrower than OOL	**2**
2	Metapectal-propodeal disc with one metapostnotal median carina and one pair of first metapostnotal lateral carinae	***Laeliustricuspis* sp. nov. (Korea)**
–	Metapectal-propodeal disc with more than five metapostnotal carinae	**3**
3	Metapectal-propodeal disc with five metapostnotal carinae	**4**
–	Metapectal-propodeal disc with seven metapostnotal carinae	8
4	Mandible with four teeth; metapectal-propodeal disc longer than wide	**5**
–	Mandible with five teeth; metapectal-propodeal disc wider than long	**7**
5	First metapostnotal lateral carinae on metapectal-propodeal disc not reaching transverse posterior carina of metapectal-propodeal complex	***Laeliussinicus*Xu et al., 2003 (China)**
–	First metapostnotal lateral carinae on metapectal-propodeal disc reaching transverse posterior carina of metapectal-propodeal complex	**6**
6	WF less than 1.5× LE; mesoscutum with notaulus; propodeal declivity with median carina	***Laeliusjilinensis* Lim & Lee, 2010 (China, S. Korea)**
–	WF more than 1.5× LE; mesoscutum without notaulus; propodeal declivity without median carina	***Laeliusafores* sp. nov. (Korea)**
7	Mesoscutum without notaulus; second metapostnotal lateral carinae reaching basal one fourth of metapectal-propodeal disc	***Laeliusyamatonis* Terayama, 2006 (Japan, Korea)**
–	Mesoscutum with distinct notaulus; second metapostnotal lateral carinae reaching basal three fourth of metapectal-propodeal disc	***Laeliussulcatus* sp. nov. (Korea)**
8	Head distinctly wider than long; pedicel 2.0× as long as wide	***Laeliusnaniwaensis* Terayama, 2006 (Japan)**
–	Head as long as wide; pedicel less than 1.5× as long as wide	**9**
9	Metapectal-propodeal disc with one metapostnotal median carina and three pairs of metapostnotal lateral carinae	**10**
–	Metapectal-propodeal disc with one metapostnotal median carina and four pairs of metapostnotal lateral carinae	***Laeliusyokohamensis* Terayama, 2006 (Japan)**
10	Legs reddish brown including coxa; first metapostnotal lateral carinae on metapectal-propodeal disc connected to metapostnotal median carina near transverse posterior carina of metapectal-propodeal complex	***Laeliusnigrofemoratus* Terayama, 2006 (Japan)**
–	Legs reddish brown except coxa and femora black; first metapostnotal lateral carinae parallel to metapostnotal median carinae	***Laeliusatratus* sp. nov. (Korea)**

## ﻿Discussion

Since Ashmead established the genus *Laelius* in 1893, a total of 68 species have been described. Notably, 50% of the valid species (33 species) have been reported since 2000, reflecting recent discoveries facilitated by the exploration of diverse diagnostic characteristics (Vikberg and Koponen 2005; Barbosa and Azevedo 2009, 2011, 2014; Lim et al. 2010b; Marques Jr. et al. 2023).

The presence and relative ratio or length of the 2r-rs&Rs vein of the forewing stand out as the most useful characteristics in the taxonomy of *Laelius* (Marques Jr. et al. 2023). However, in the case of the four new species in the present study, they commonly exhibit relatively similar lengths of the 2r-rs and Rs veins.

Barbosa and Azevedo (2011) conducted cladistic analyses based on 108 female structural characters, revealing that the genus is supported by six autapomorphies: a body with thick setae present; a straight profile of the median clypeal carina; an incomplete anterior extension of the median clypeal carina; an angled anterior corner of the pronotal disc; three distal hamuli on the hindwing; and tergum II longer than the others. The four new species from the present paper, namely *L.afores* sp. nov., *L.atratus* sp. nov., *L.tricuspis* sp. nov., and *L.sulcatus* sp. nov., commonly exhibit these autapomorphies suggested by Barbosa and Azevedo (2011). Additionally, the ratios of POL, OOL, WF, LE, antennal segments, number of median discal carinae, number of mandibular teeth, and the presence of a median carina on the propodeal declivity are useful characteristics for the delimitation of new species.

In Colombo et al. (2022), a more recent study of the phylogenetic relationships of Epyrinae, a clade of six genera, including *Laelius*, was found to be monophyletic. Furthermore, a clade of four genera, namely *Anisepyris*, *Austrepyris*, *Chlorepyris*, and *Laelius*, which commonly share the characteristic of the mesoscuto-scutellar sulcus being well impressed and incurved medially, is sister to the remaining Epyrinae.

*Laelius* species exhibit weak sexual dimorphisms and share many common characteristics except for genitalia structures (Azevedo et al. 2018). Since most species of *Laelius* worldwide were described based on females and do not include enough species for cladistic studies on male genitalia, it is necessary to obtain many male samples for studies on the genus. Additionally, Colombo et al. (2022) analyzed nine species of *Laelius*, mostly from the Nearctic and Neotropical regions, except for one species from the Palaearctic region (Israel), for constructing Epyrinae phylogeny. Indeed, a clade including *Laelius* was supported as the sister group against the other genera of Epyrinae, necessitating further exploration of the phylogenetic relationships among the genera in the clade containing *Laelius* with more diverse species from various zoogeographic regions, including Eastern Asia.

In the present paper, four additional *Laelius* species from the Korean Peninsula, part of Far Eastern Asia, were described. Consequently, five *Laelius* species have been recorded in the nation, which represents higher species diversity compared to neighboring countries such as Japan (4), China (1), and Far Eastern Russia (1). This leads us to speculate that there are more unknown species in the Eastern Palaearctic region.

## Supplementary Material

XML Treatment for Laelius

XML Treatment for Laelius afores

XML Treatment for Laelius atratus

XML Treatment for Laelius sulcatus

XML Treatment for Laelius tricuspis

## ﻿Additional information

### Author ORCIDs

Jongok Lim https://orcid.org/0000-0001-5700-3024

Seunghwan Lee https://orcid.org/0000-0003-3026-5328

### Data availability

All of the data that support the findings of this study are available in the main text.
